# Engagement with a youth violence intervention programme is associated with lower re-attendance after violent injury: A UK major trauma network observational study

**DOI:** 10.1371/journal.pone.0292836

**Published:** 2023-10-18

**Authors:** Edward A. Dickson, Lauren Blackburn, Miriam Duffy, David N. Naumann, Adam Brooks

**Affiliations:** 1 East Midlands Major Trauma Centre, Nottingham University Hospitals NHS Trust and University of Nottingham, Queen’s Medical Centre, Nottingham, United Kingdom; 2 Academic Department of Military, Surgery and Trauma, Royal Centre for Defence Medicine, University Hospitals Birmingham NHS, Foundation Trust, Birmingham, United Kingdom; Yale University, UNITED STATES

## Abstract

The hospital based Redthread Youth Violence Intervention Programme (YVIP) utilises experienced youth workers to support 11–24 year olds following an episode of violent injury, assault or exploitation who present to the Emergency Department (ED) at the East Midlands Major Trauma Centre (MTC), Nottingham, UK. The YVIP aims to promote personal change with the aim of reducing the incidence of further similar events. We conducted a retrospective, observational, cohort study to examine the association between engagement with the YVIP and re-attendance rates to the ED following a referral to Redthread. We also examined factors associated with engagement with the full YVIP. We found that 573 eligible individuals were referred to the YVIP over two years. Assault with body parts 34.9% (n = 200) or a bladed object 29.8% (n = 171) were the commonest reason for referral. A prior event rate ratio (PERR) analysis was used to compare rates of attendance between those who did and did not engage with the full YVIP. Engagement was associated with a reduction in re-attendances of 51% compared to those who did not engage (PERR 0.49 [95% 0.28–0.64]). A previous attendance to the ED by an individual positively predicted engagement. (OR 2.82 [95% CI 1.07–7.42], P = 0.035). A weekend attendance (OR 0.26 [0.15–0.44], P<0.001) and a phone call approach (OR 0.25 [0.14–0.47], P = 0.001), rather than a face-to-face approach by a Redthread worker, negatively impacted engagement. In conclusion, assaults with or without a weapon contributed to a significant proportion of attendances among this age group. The Redthread YVIP was associated with reduced rates of re-attendance to the East Midlands MTC among young persons who engaged with the full programme.

## Introduction

Youth violence and recidivism present a major healthcare and socioeconomic burden to the UK according to the Government Home Office [1]. Without intervention repeat attendance rates to hospital following violent injury are estimated to occur in 22–44% of individuals [2]. Young persons seeking medical attention for a violent injury are twice as likely to re-attend the Emergency Department (ED) for a further episode than those attending for non-violent reasons [3] and early exposure to violence and other key risk factors may be linked to serious violence in later life [4]. In particular, evidence suggests that 20% of males convicted of homicide in later life had been linked to offences when they were under the age of 13 [5].

Violent incidents and the effects of violence can result in substantial financial expense for healthcare systems [6]. Youth violence carries a significant cost to both victims and perpetrators either related to healthcare services, lost output due to injury, policing costs, or victim services [7]. Studies from North America have shown that hospital-based violence intervention programmes can decrease violent injury recidivism [8, 9]. Therefore, a programme that reduces youth violence may be a valuable tool for the health and wellbeing of individuals, for healthcare services and society. Whilst evidence from the UK was historically lacking, there has now been an emergence of data from UK based studies [10]. However, many of these reports are limited by low patient numbers and the heterogeneity of interventions and reporting has impacted their generalisability.

The Redthread Youth Violence Intervention Programme (YVIP) was established at the East Midlands Major Trauma Centre (MTC) in Nottingham in 2018 [11]. The Redthread theory of change has been previously described [10]. It aims to offer intervention for young persons following a traumatic or adverse event at the earliest opportunity. The programme centres around the concept of the ‘teachable moment’, defined as a “naturally occurring life transition or health event thought to motivate individuals to spontaneously adopt risk-reducing health behaviours” [12]. This has been used widely as a mechanism to promote change across a range of health and social care fields [13]. The Redthread YVIP also builds its foundations on the Health Belief Model [14] which explains that engagement in health promoting behaviour or lifestyle change is often subject to a person’s in-built beliefs surrounding the benefits or barriers to change. Further, the model also describes the need for a trigger or catalyst to promote this change [15]. Much of the efforts by Redthread youth workers are focussed around supporting young persons by building trust and rapport to positively emerge from an adverse incident. Ongoing engagement reinforces this change to prevent a relapse into old behaviours. This concept further draws on aspects of the Transtheoretical Model [16] which explains the process of intentional behaviour change including the stages prior to a behaviour change, notably pre-contemplation (not ready), contemplation (getting ready) and preparation (ready).

This study aimed to evaluate the association between engagement with the Redthread YVIP and further episodes of violent injury requiring re-attendance to hospital for young persons referred to the service. We hypothesised that engagement with the YVIP would be associated with a reduction in involvement in future violence leading to lower rates of re-injury and re-attendance to the ED.

## Methods

### Study design and setting

A retrospective observational study was undertaken to include all young persons referred to the East Midlands Redthread YVIP from March 2018 –March 2020. Eligibility for the YVIP included age 11–24 years and an ED attendance due to violent injury, sexual assault, exploitation, substance misuse or self-harm.

### Redthread referral and involvement

Redthread referrals are sent at any time of day and can come directly from clinicians treating these young persons or patients can be identified via the hospital database by Redthread youth workers. To capitalise on the concept of the ‘teachable moment’ [17]. Redthread youth workers may attend the young person immediately after arrival to the ED in a trauma scenario. After initial contact, with consent, Redthread workers seek to continue this support on the ward and following discharge from hospital to foster and maintain positive change in the young person’s life.

### Subsequent engagement with Redthread

Engagement with the programme is voluntary and individuals require varying levels of support. Following verbal consent to the YVIP, an assessment and safety plan is formulated encompassing a holistic approach to a young person’s physical, mental, and social needs. This may involve simple and immediate measures (‘crisis support’) such as support with emergency accommodation, safe travel, advocacy and signposting with specialist hospital and community agencies. Or they could extend to engagement in a full, longer programme, usually up to 12 weeks or longer. Redthread youth workers undertake a comprehensive risk and needs assessment of the young person with a co-produced action plan. They include involvement with safeguarding teams, support with navigating systems (e.g., criminal justice or welfare), advocacy work with statutory partners and community agencies, and relational referrals for ongoing specialist support. It can include casework around healthy relationships and managing difficult emotions, support to (re-)engage with education, training and employment, securing longer term accommodation or accessing mental health or substance misuse support.

### Data collection

The records of all included individuals were examined for hospital attendances two years prior to their referral to Redthread. Data collection was undertaken using hospital electronic records and anonymised data held by the Redthread team in line with their data sharing agreement with the NHS Trust. Only re-attendances to the ED at Queens Medical Centre (the MTC), Nottingham, UK were available for analysis. Patients were not contacted to provide additional information to the study team. An Injury Severity Score (ISS) was calculated for each patient attendance during the study period. A higher ISS indicates more severe injury.

### Ethical approval

The study was approved by the London–Bromley NHS Research Ethics Committee (ref 20/LO/0691) and the Health Research Authority. Consent was sought to access the Trauma Audit and Research Network (TARN) database to determine whether these patients had been referred to Redthread, if eligible [18].

### Patient and public involvement

The design of the study was conducted in consultation with key stakeholders including hospital clinicians and local commissioners to the ensure outcomes and data generated would guide future policy and funding for the YVIP. In addition, the views of young persons including those who had been involved in the YVIP and members of the Redthread youth-worker team both locally and nationally were sought.

### Outcome measures

The primary outcome was rate of re-attendance to the ED, calculated as events per 100 person years. Pre-defined secondary outcomes included re-attendance rates of all YVIP referrals, engagement with the Redthread YVIP, length of stay in hospital, surgical intervention and mortality. Re-attendance was defined as an individual returning to the emergency department at a future timepoint due to another incident of violent injury, sexual assault, exploitation, substance misuse or self-harm.

### Data analysis

#### Data presentation

Categorical data are presented with number and percentages. Continuous data are presented with mean and standard deviation (SD) for normally distributed data, and median and inter-quartile ranges (IQR) for non-normal data. Data distribution was examined visually for skewedness. Data were analysed using SPSS (Version 27.0. Armonk, NY: IBM Corp). A p-value of <0.05 was considered statistically significant for all analyses.

#### Primary analyses

The primary outcome (re-attendance in ED) was compared between those who engaged with the Redthread YVIP and those who did not, as well as before and after engagement with the YVIP. We used a prior event rate ratio (PERR) analysis [19] to compare the change in rate of attendance before and after referral to the YVIP. The PERR model aims to replicate the conditions of a randomised controlled trial. The PERR analyses the outcomes of a group of individuals exposed to the intervention (i.e., the YVIP) within a certain timeframe compared to those unexposed to the intervention. The PERR assumes that the hazard ratio of those receiving or not engaging with the YVIP for a specific outcome (re-attendance) before the start of the study period reflects the combined effect of all confounders (both measured and unmeasured) independent of the impact of the YVIP [20]. The benefit of the PERR lies in its ability to account for these unmeasured confounding factors when comparing rates of attendance between the two groups. The PERR was estimated by the ratio of two unadjusted hazard ratios: the unadjusted hazard ratio for re-attendance during the time since the YVIP was introduced to the engaged group vs the non-engaged group (HR.post) and the unadjusted hazard ratio for re-attendance before the YVIP was introduced for the engaged group vs non-engaged group (HR.prior). The PERR is then calculated from the ratio of HR.post/HR.prior. Hazard ratios were calculated from the event rates for engagers and non-engagers. Both univariable and multivariable logistic regression were used to compare the odds of engagement with the YVIP based on patient characteristics and factors related to the mode and timing of referral.

#### Subgroup analyses

Subgroup analysis was planned for individuals living at addresses only within the Nottingham City and Nottinghamshire County area, to mitigate the potential limitation of a person re-attending at another hospital in the event of a more minor injury.

## Results

### Study patient characteristics

**Fig 1:**
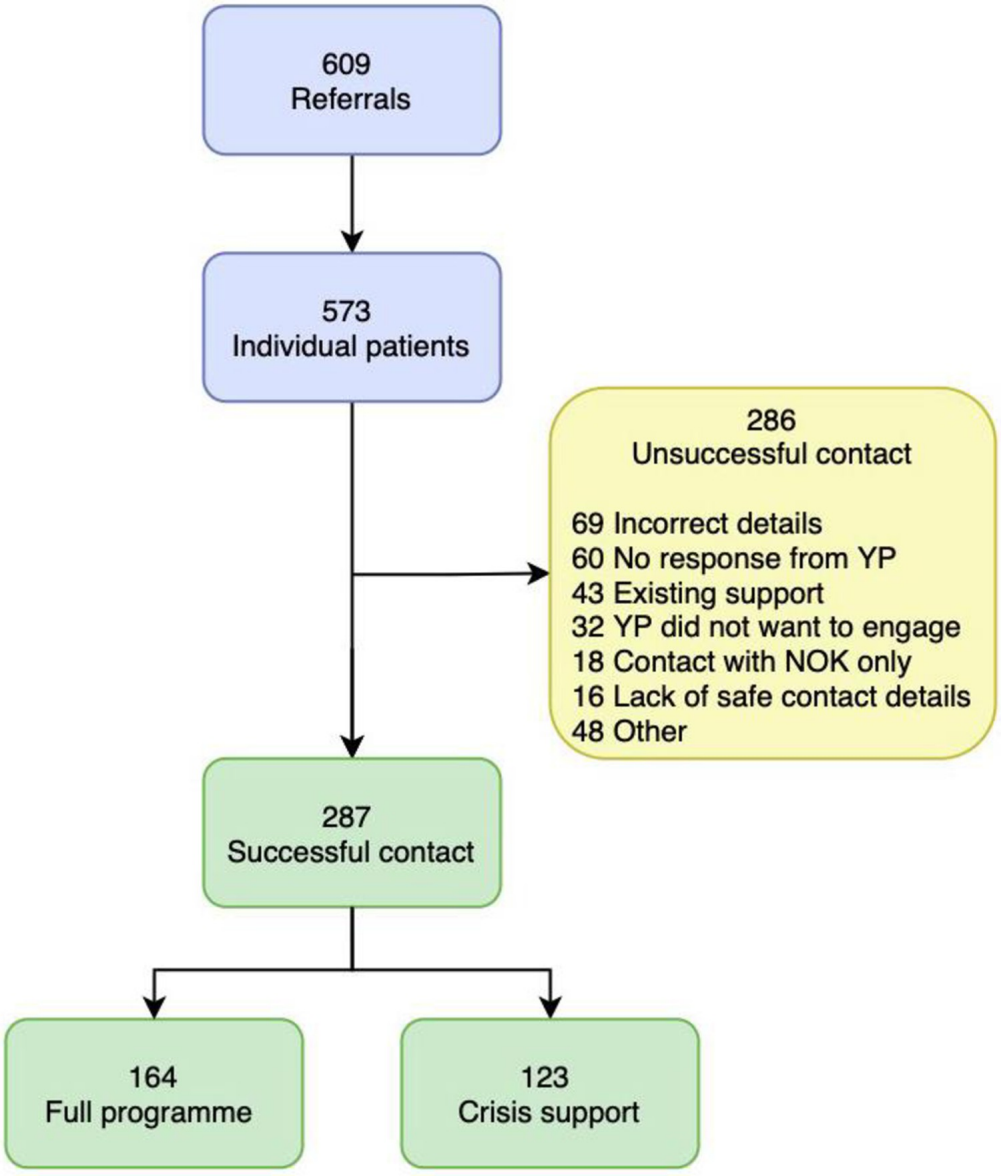
Flow diagram for Redthread YVIP referrals, YP, young person; NOK, next of kin.

From March 2018 to March 2020 a total of 647 referrals to the YVIP were made. Of these, 609 referrals were eligible for inclusion, accounting for 573 individual patients. Among all individuals referred 77% (n = 439) were male and 68.1% (n = 390) of referrals were in the 15–21 age bracket. Redthread made successful initial contact with 287/573 of referred patients. Among those successfully contacted 164/287 (57%) engaged in a full programme of support and 123/287 (43%) received crisis support limited to their index attendance. Non-engagement occurred in 286 cases (Fig 1). Therefore, in relation to our primary outcome measure, the study cohort consisted of 164/573 (29%) who engaged in the full programme (“engagers") and 409/573 (71%) who did not engage in the full programme (“non-engagers”). Across all index attendances to hospital, median ISS was 2 (IQR 1–4, range 1–50). Among those admitted median ISS was 9 (IQR 5–18, range 1–50) for engagers and 2 (IQR 1.75–4, range 1–34) for non-engagers.

### Hospital episodes

Discharge directly from the ED occurred in 318/409 (78%) of non-engagers and 104/164 (63%) of engagers. For those YVIP engagers admitted to hospital from ED at their index attendance, the median length of stay was 3 days (IQR 1–6, range 1–37). For non-engagers who were admitted, the median length of stay was 5 days (IQR 2–9, range 1–34). Among non-engagers re-attending after their index attendance 8 were admitted with a median length of stay of 5 days (IQR 3.5–4.5, range 2–14) and 3 needed surgical management of their injuries. Indications for attendance triggering a YVIP referral are outlined in Table 1. Index attendances triggering referral to the YVIP were clustered around the weekend (Friday-Sunday) with 55.1% (n = 330) occurring over these 3 days. Assault related injuries were evenly shared between weekday and weekend accounting for 87.2% (n = 287) and 88.4% (n = 290) of attendances on these days, respectively. The timing of index attendances to the Emergency Department saw the lowest attendance rate at 0700 hours followed by a steady upward trend towards a peak at 1700 hours.

**Table 1:** Primary indication for attendance triggering referral to the Redthread YVIP at index attendance for all patients and compared between engagers and non-engagers. All data are presented as percentages and frequencies in parentheses.

Mechanism	Total (n = 573)	Engaged in full programme (n = 164)	Not engaged in full programme (n = 409)
**Assault**			
Blunt object	7.5% (n = 43)	6.7% (n = 11)	7.8% (n = 32)
Burns	0.9% (5)	1.2% (2)	0.7% (3)
Body parts	34.9% (200)	31.1% (51)	36.4% (149)
Glass/Bottle	3.1% (18)	1.8% (3)	3.7% (15)
Firearm	2.1% (12)	3% (5)	1.7% (7)
Knife or bladed object	29.8% (171)	33.5% (55)	28.4% (116)
Vehicle	0.5% (0.5)	0	0.7% (3)
**Sexual assault**	1.7% (10)	3.7% (6)	1.0% (4)
**Exploitation**	0.5% (3)	1.2% (2)	0.2% (1)
**Mental Health**			
Intentional overdose	2.6% (15)	2.4% (4)	2.7% (11)
Self-harm	2.1% (12)	1.8% (3)	2.2% (9)
Suicidality	1.0% (6)	1.2% (2)	1.0% (4)
Other	1.4% (8)	0.6% (1)	1.7% (7)
**Substance**			
Alcohol	1.6% (9)	2.4% (4)	1.2% (5)
Drugs	4.0% (23)	6.1% (10)	3.2% (13)
**Accident**			
Self-inflicted	3.0% (17)	2.4% (4)	3.2% (13)
Road traffic collision	0.9% (5)	0	1.2% (5)
**Illness/safeguarding**	2.3% (13)	0.6% (1)	2.9% (12)

Across the entire study period 706 individual incidents of violent injury or assault occurred. From these, 59% (n = 415) of incidents had sufficient data on hospital electronic records to accurately map their location. Distance from home to location of incident was mapped in all cases with sufficient data. Mean (SD) distance of an incident occurring away from a home address was 1.78 (1.3) kilometres (range 0.2–257km).

### Hospital re-attendance

#### All re-attendances

Across the entire cohort 20.2% (n = 116) had attended the ED in the 2 years prior to referral to the YVIP (full programme engagers 29.9% [n = 49/164], non-engagers 16.4% [n = 67/409]. Among these individuals the median number of visits was 1 for both groups (engagers range 1–22 visits; non-engagers 1–5). Attendance rates had dropped from 29.9% pre intervention to 18.3% (n = 30/164) post intervention for individuals engaging with the full YVIP (Table 2). For non-engagers rates of attendance increased from 16.4% prior to their YVIP referral to 18.1% (n = 74/409) after non-engagement. This translated to an absolute reduction in the percentage of individuals re-attending the ED of 11.6% for the engaged group and an increase of 1.7% for non-engagers. All re-attendances were recorded after approach to enrol in the YVIP. For those with a history of attendances after approach the median number of visits was 1 for both groups (engagers range 1–10 visits; non engagers 1–7) (S1 Fig).

**Table 2:** Event rates and unadjusted hazard ratios for Emergency Department attendances with 95% confidence intervals among all patients referred to the YVIP who engage and do not engage with the Redthread full YVIP presenting with a violent injury or assault. A proportion of not engaged include those who received crisis support only (n = 123).

Parameter	Engaged in full programme	Not engaged in full programme
No of patients	164	409
**Before approach by Redthread**		
No (%) patients with prior attendances in 2yr before approach	49 (29.9%)	67 (16.4%)
Incidence of attendances per 100 person years (95% CI)	30.5 (24.6–37.8)	12.4 (10.1–15.2)
Unadjusted hazard ratio (engaged/non-engaged) (95% CI)	2.45 (2.2 to 3.67)	
**After approach by Redthread**		
No (%) patients with attendances after approach up to database lock	30 (18.3%)	74 (18.1%)
Incidence of attendances per 100 person years (95% CI)	25.8 (19.8–33.7)	26.6 (22.1–31.9)
Unadjusted hazard ratio (engaged/non-engaged) (95% CI)	0.96 (0.67–1.40)	
Prior event rate ratio* (95% CI)	0.39 (0.22–0.51)	

#### Following violence & assault

For those with an index attendance related to violence or assault full engagement with the YVIP led to a drop in recidivism from 27.3% to 13.6% giving a relative reduction of 50.2%. In contrast, for the non-engaged group recidivism remained entirely consistent at 15.3%. The PERR was 0.37 (0.19–0.58) for the rate of attendances after engaging with the YVIP specifically after a violent injury or assault (Table 3). A similar reduction was seen among all referrals to the YVIP and those specifically living in Nottingham or Nottinghamshire postcode (S1 Table).

**Table 3:** Event rates and unadjusted hazard ratios for Emergency Department attendances due to violent injury or assault recidivism (n = 471) with 95% confidence intervals among those eligible for the YVIP who engage and do not engage with the Redthread full. A proportion of not engaged include those who received crisis support only (n = 123).

Parameter	Engaged in full programme	Not engaged in full programme
Number of patients	139/471	332/471
**Before approach by Redthread**		
Number (%) patients with prior attendances in 2yr before approach	38/139 (27.3%)	51/332 (15.3%)
Incidence of recidivism per 100 person years (95% CI)	18.7 (14–25.1)	10.3 (8.1.-13)
Hazard ratio (engaged/non-engaged) (95% CI)	1.81 (1.31–2.50)	
**After approach by Redthread**		
Number (%) patients with attendances after approach up to database lock	19/139 (13.6%)	55/332 (15.3%)
Incidence of recidivism per 100 person years (95% CI)	13.6 (9.2–20.1)	20.2 (16.2–25.2)
Hazard ratio (engaged/non-engaged) (95% CI)	0.67 (0.44–1.07)	
Prior event rate ratio* (95% CI)	0.37 (0.19–0.58)	

### Factors associated with engagement

The only patient factor demonstrating a relationship with odds of engagement in univariate analysis was age, but this was not significant on multivariable analysis (Table 4). On univariate analysis a face-to-face referral and approach offered the highest odds of engagement (Table 5). Previous attendances by the young person and multiple prior referrals to the YVIP were also shown to increase odds of engagement. A weekend attendance, approach via a phone call or text message/letter and delays of >24 hours from attendance to referral decreased odds of engagement. Odds ratios which remained statistically significant for engagement with the YVIP after adjustment on multivariable analysis included day of attendance, multiple previous referrals, and method of approach (Table 5).

**Table 4:** Univariable and multivariable logistic regression analysis of the odds ratios (OR) with 95% confidence interval (95% CI) for engagement with the YVIP according to patient characteristics.

	Unadjusted OR (95% CI) (n = 490)	*p*-value	Adjusted OR (95% CI) (n = 303)	*p*-value
**Gender**				
Male	1		1	
Female	1.16 (0.74–1.84)	0.503	0.98 (0.40–2.42)	0.980
**Age**				
11–15	1		1.00	
16–20	0.48 (0.28–0.83	0.008	0.53 (0.23–1.22)	0.137
20–24	1.74 (0.55–2.64	0.864	0.96 (0.41–2.23)	0.937
**Ethnicity**				
White^a^	1		1	
Black^b^	1.57 (0.69–3.72)	0.298	1.99 (0.73–5.38)	0.173
Asian^c^	0.66 (0.24–1.81)	0.426	0.83 (0.28–2.48)	0.745
Mixed^d^	0.64 (0.20–2.00)	0.443	0.49 (0.14–1.78)	0.281
Other^e^	0.50 (0.14–1.84)	0.296	0.28 (0.56–1.45)	0.129
**Home Location**				
Nottingham City	1		1	
Nottinghamshire	1.26 (0.78–2.04)	0.330	0.87 (0.37–2.08)	0.765
Derbyshire	1.16 (0.59–2.30)	0.658	1.39 (0.47–4.13)	0.545
Leicestershire	0.63 (0.26–1.53)	0.314	0.87 (0.26–2.91)	0.822
Lincolnshire	1.63 (0.35–7.47)	0.527	0.90 (0.49–16.92)	0.947
Other	0.96 (0.40–2.32)	0.941	0.97 (0.25–3.78)	0.971
**Reason for Referral**				
Assault	1		1	
Domestic abuse	1.31 (0.64–2.71)	0.451	2.89 (0.74–11.32)	0.126
Risk of harm^f^	1.93 (0.89–4.18)	0.094	1.43 (0.39–5.23)	0.206
Other	1.48 (0.41–5.37)	0.544	1.40 (0.20–9.49)	0.731
**SDS**				
1–3	1		1	
4–7	0.73 (0.46–1.17)	0.190	1.15 (0.52–2.55)	0.727
8–10	0.67 (0.36–1.29)	0.679	1.46 (0.49–4.33)	0.493

SDS, Social deprivation score (1-most deprived, 10-least deprived)

^a^White British/Irish

^b^Black British/Black Caribbean/Black African/ Black Other

^c^Asian British/Asian Bangladeshi/Asian Pakistani/Asian Other

^d^Mixed White & Black British/Caribbean/African/Asian

^e^European/Other.

^f^ Includes gang involvement and exploitation.

**Table 5:** Univariable and multivariable logistic regression analysis of the odds ratios (OR) with 95% confidence interval (95% CI) for engagement with the YVIP according to method and type of referral.

	Unadjusted OR (n = 490)	p-value	Adjusted OR ^a^ (n = 388)	p-value
**Mode of referral**				
Face to face	1		1	
Electronic	0.47 (0.25–0.89)	0.02	0.79 (0.35–1.80)	0.581
Hospital database	0.25 (013–0.46)	<0.001	0.49 (0.56–4.28)	0.520
Telephone	0.65 (0.32–1.32)	0.238	0.64 (0.27–1.53)	0.316
Paper^b^	-	-	-	-
**Weekend attendance**				
No	1		1	
Yes	0.22 (0.14–0.34)	<0.001	0.26 (0.15–0.44)	<0.001
**Referrer**				
Doctor	1		1	
Nurse	0.68 (0.38–1.22)	0.197	0.68 (0.32–1.41)	0.295
Safeguarding team	1.11 (0.25–4.85)	0.893	0.93 (0.16–5.39)	0.941
Self	0.26 (0.10–0.65)	0.004	0.32 (0.03–3.34)	0.346
Hospital database	0.33 (0.17–0.63	0.001	0.91 (0.10–8.21)	0.930
Other	0.36 (0.07–1.98)	0.245	0.14 (0.12–1.51)	0.104
**Discharge outcome**				
Admitted	1		1	
Discharged from ED	0.44 (0.29–0.67)	<0.001	0.84 (0.45–1.54)	0.836
Self-discharge/did not wait	0.18 (0.40–0.84)	0.029	0.21 (0.02–2.29)	0.210
**Previous attendances**				
Yes	1		1	
No	0.59 (0.38–0.94)	0.026	0.63 (0.34–1.15)	0.627
**Method of contact**				
Face to face	1		1	
Phone call	0.21 (0.13–0.32)	<0.001	0.25 (0.14–0.47)	0.001
Text message or letter	0.17 (0.36–0.78)	0.023	0.18 (0.33–0.96)	0.045
**Time from attendance to referral**				
≤24 hours	1		1	
>24 hours	0.38 (0.21–0.71)	0.002	0.83 (0.38–1.79)	0.642
**Multiple referrals**				
No	1		1	
Yes	3.39 (1.59–7.24)	0.002	2.82 (1.07–7.42)	0.035

ED, Emergency Department

^a^adjusted for all other variables within table.

^b^paper referrals dropped due to predicting failure perfectly.

### Secondary outcomes

There were no deaths due to further traumatic injury or violent assault in the entire cohort of Redthread referrals. Surgical intervention was required in 13.4% (n = 22) patients. For the 25 further attendances registered by young persons after engagement with the YVIP just one patient required emergency surgical management of their injuries.

## Discussion

In our study of 573 patients referred to the Redthread YVIP following violent injury at a large UK MTC, we found that engagement with the full YVIP was associated with a reduction of 50–60% in rates of re-attendance when compared those who did not engage. Factors that significantly increased the odds of engagement included a face-to-face approach to the young person, recurrent referrals, and a weekday attendance. For those who engaged in a full programme of support 18% of individuals subsequently re-attended which falls just below the estimate of 22–44% in the literature [2]. We chose to report attendances according to the event rate of those who engage and do not engage with the YVIP as this offers an estimation of impact resource utilisation. Prior attendance rates before referral to the YVIP were around 2.5-fold higher in those who later went on to engage with the full YVIP when compared to those who did not, suggesting that these groups may differ in their underlying characteristics. These findings also suggest that recurrent previous attendances may in fact be a catalyst for engagement with the YVIP linking back to the notion of the ‘teachable moment’. One of our most striking observations was the increase in further admissions for those choosing not to engage with the YVIP. This serves to illustrate the relapsing trends of youth violence in young persons who lack support for change [21].

The difference in outcomes for those re-attending is also important to consider, particularly with regards to subsequent intervention and the cost of ongoing medical care. The non-engaged cohort had a greater frequency of inpatient admission. (engaged n = 5, non-engaged n = 8) and ISS was also marginally higher in this group. ISS scores correlate with hospital costs with estimates ranging from £6000 for lower scores to over £16000 for the most severely injured individuals according to TARN data from England and Wales [22]. Therefore, our observation of a reduction in ISS scores for the engaged cohort is likely to have offered cost savings at an institutional level. Patterns of attendance in our cohort followed trends seen studies of youth violence in other major UK cities such as London [23] and Birmingham [24] with evidence suggesting a high volume of assault, particularly with a knife or blade, and the majority of incidents occurring close to the victims home. Interestingly, attendances peaked in the late afternoon suggesting that injury linked to the night-time economy may be less of a dominant factor. These findings mirror those by Vulliamy et al. who observed a similar spike in incidents after the school day finished [23]. This evidence should act to further support for the ongoing work by Redthread, and the need to continue to fund the provision of an out of hours service given that these assaults may not require input from Redthread until the early evening.

It is evident from both univariable and multivariable analysis that there were important factors predicting the odds of success when referring a young person to the YVIP. We hypothesise that a lack of opportunity for face-to-face discussion with a young person or an attendance at a weekend may miss the opportunity of a ‘teachable moment’. Indeed, those discharged directly from ED without a face-to-face meeting with a Redthread youth worker had less than half the odds of engagement with the YVIP compared to those who were admitted. Interestingly, multiple referrals appeared to significantly impact the odds of engagement with those who had been referred on more than one occasion seeing engagement increase nearly 3-fold. This emphasises the importance of continuing to offer support to young persons who recurrently attend the emergency department.

Across our analysis of demographic factors affecting odds of engagement it was surprising to conclude that none of the variables analysed in our multivariable model affected chance of engagement. This analysis was subject to considerable missing data, particularly relating to ethnicity, which may have impacted the generalisability of results. Further analysis with a higher sample size, perhaps collating nationwide data from Redthread may offer a more robust conclusion.

### Limitations

There were a number of challenges and limitations faced during this study. Firstly, all data obtained was retrospective and therefore all analyses are subject to the accuracy of this. In addition, as this study involved analysis of data at a single site, there may have been re-attendances at peripheral hospitals. However, to partially mitigate this, we have offered an analysis solely of Nottingham City/County residents who would attend the MTC even for more limited injuries. Events may also have occurred, such as minor assaults, where medical attention was not sought and therefore not reported in this study. Therefore, the societal impact of the YVIP on youth violence may have been under reported. Nevertheless, this study does offer data on the impact of the YVIP on healthcare resource at a high-volume UK Major Trauma Centre. Also, it is important to note our group of non-engaged individuals did, in some cases, receive crisis support and therefore the association of not receiving the intervention may have been underestimated in this group. The main limitation of the PERR analysis is that prior events may possibly modify the risk of subsequent exposure. Whilst we can be sure that individuals had not been exposed to the YVIP they may have been exposed to similar programmes elsewhere through statutory services. This could therefore lead to bias of the PERR adjustment. Indeed, simulation studies have noted increased bias of the PERR in cases where prior events influence the probability of exposure to the intervention even when there is no exposure effect on the outcome [25]. In our study the probability of exposure to the YVIP is strongly influenced by prior event, e.g., incidents of violent injury, which could be seen as a limitation of this analysis.

There are also limitations to the YVIP itself. Eligibility for the programme must be limited by certain criteria, such as age. As such, the programme is unable to modify risk factors which stem from early childhood. For instance, It is known that factors such as physical and sexual abuse during childhood have shown an association with future violent behaviour among victims [26]. Those being referred to the YVIP at the upper age limit may have deep seated risk factors which are challenging to address.

To conclude, this study has served to highlight the impact of the Redthread YVIP at a UK major trauma centre. According to our analysis full engagement with the YVIP was associated with a reduction in re-attendances to the Emergency Department at the MTC.

## Supporting information

S1 Fig Frequency of further attendances after approach by Redthread according to non-engagement and engagement. (DOCX)

S1 Table Event rates and unadjusted hazard ratios for Emergency Department attendances with 95% confidence intervals among those eligible for the YVIP who engage and do not engage with the Redthread YVIP living in a Nottingham City or Nottinghamshire postcode. (DOCX)
