# Supplementary material for: Engagement with a youth violence intervention programme is associated with lower re-attendance after violent injury: A UK major trauma network observational study
Source: PLoS One. 2023 Oct 18;18(10):e0292836. doi: 10.1371/journal.pone.0292836 (PMC10584091; doi:10.1371/journal.pone.0292836)
Supplement: S1 Fig — (DOCX) [file pone.0292836.s001.docx]

Supplementary figure 1 – Frequency of further attendances after approach by Redthread according to non-engagement and engagement.
