# Supplementary material for: Engagement with a youth violence intervention programme is associated with lower re-attendance after violent injury: A UK major trauma network observational study
Source: PLoS One. 2023 Oct 18;18(10):e0292836. doi: 10.1371/journal.pone.0292836 (PMC10584091; doi:10.1371/journal.pone.0292836)
Supplement: S1 Table — (DOCX) [file pone.0292836.s002.docx]

Supplementary table 1 - Event rates and unadjusted hazard ratios for Emergency Department attendances with 95% confidence intervals among those eligible for the YVIP who engage and do not engage with the Redthread YVIP living in a Nottingham City or Nottinghamshire postcode.

| **Parameter** | **Engaged in full programme** | **Not engaged in full programme** |
| --- | --- | --- |
| No of patients with a Nottingham City or Nottinghamshire postcode | 136 | 322 |
| **Before approach by Redthread** |  |  |
| No (%) patients with prior attendances in 2yr before approach | 44 (32%) | 59 (18.3%) |
| Incidence of attendances per 100 person years (95% CI) | 25.1 (19.4-32.7) | 13.2 (10.5-16.5) |
| Unadjusted hazard ratio  (engaged/non-engaged) (95% CI) | 1.90 (1.38 to 2.60) | |
| **After approach by Redthread** |  |  |
| No (%) patients with attendances after approach up to database lock | 29 (21%) | 60 (18.6%) |
| Incidence of attendances per 100 person years (95% CI) | 23 (17.2-30.8) | 25.6 (20.9-31.3) |
| Unadjusted hazard ratio (engaged/non-engaged) (95% CI) | 0.89 (0.62-1.29) | |
| Prior event rate ratio* (95% CI) | 0.46 (0.29-0.65) | |
